# Controlled Synthesis of N-Doped Hierarchical Porous Carbon Spheres Through Polydopamine for CO_2_ Adsorption and High-Performance Supercapacitors

**DOI:** 10.3390/molecules30132747

**Published:** 2025-06-26

**Authors:** Xiaoqi Jin, Jinlong Ge, Zhong Wu, Linlin Zhu, Mingwen Xiong, Jiahui Qi, Chengxiu Ruan

**Affiliations:** 1 Anhui Province Engineering Laboratory of Silicon-Based Materials, School of Materials and Chemical Engineering, Bengbu University, 1866 CaoShan Road, Longzihu District, Bengbu 233030, China; jinlongge2005@126.com (J.G.); wuzhong2018@bbc.edu.cn (Z.W.); zhulilin654@163.com (L.Z.); xiongmingwen@163.com (M.X.); 2 School of Materials and Chemical Engineering, Bengbu University, Bengbu 233030, China; 1972844150@163.com (J.Q.); 19356394837@163.com (C.R.)

**Keywords:** N-doped carbon spheres, hierarchical pore structure, CO_2_ adsorption, supercapacitor

## Abstract

Hierarchical porous N-doped carbon spheres featuring a combination of micropores, mesopores and macropores as well as tuneable properties were synthesised using dopamine as a carbon precursor and triblock copolymers (F127, P123 and F127/P123 composites) as templates via direct polymerisation-induced self-assembly. The structures and textures of these materials were characterised using X-ray diffraction, scanning electron microscopy, transmission electron microscopy, N_2_ adsorption–desorption isotherm analysis, Fourier-transform infrared spectroscopy, Raman spectroscopy and X-ray photoelectron spectroscopy. The sample synthesised at an F127:P123 molar ratio of 1:3 (NCS-FP3) exhibited the highest surface area (463 m^2^/g) and pore volume (0.27 cm^3^/g). The hydrophobic/hydrophilic molar ratios of the templates were adjusted to control the morphology of the corresponding micelles and hence the porous structures and morphologies of the carbon spheres, which exhibited high CO_2_ capture capacities (2.90–3.46 mmol/g at 273 K and 760 mmHg) because of their developed microporous structures and N doping. Additionally, NCS-FP3 exhibited an outstanding electrochemical performance, achieving a high specific capacitance (328.3 F/g at a current density of 0.5 A/g) and outstanding cycling stability (99.2% capacitance retention after 10,000 cycles). These high CO_2_ capture and electrochemical performances were ascribed to the beneficial effects of pore structures and surface chemistry features.

## 1. Introduction

Owing to their adjustable pore structure, high specific surface area, low density, excellent mechanical stability and stable electrochemical performance, porous carbon materials have been employed in diverse applications, including adsorption, catalysis, biomedicine, separation, energy storage (e.g., supercapacitors and Li-ion batteries) and sulphur hosts [1,2,3,4,5,6,7]. Previous decades have witnessed substantial advancements in tailoring the morphologies and pore structures of porous carbon materials to enhance their physicochemical properties. Amongst porous carbon materials with different (e.g., spherical, core–shell, sheet and bowl-like) particle shapes [8,9,10,11,12], hierarchical porous carbon spheres containing micropores and mesopores have attracted extensive attention as their high packing density and mechanical strength result in a short diffusion distance and low fluid resistance, enabling highly effective gas adsorption and drug delivery. Previous works by our [13] and other research groups [14,15] showed that micropores/mesopores in hierarchical porous materials exhibit remarkable synergistic effects that substantially enhance mass transport efficiency.

The performance of porous carbon spheres can be enhanced by modulating their electronic and surface properties via doping with heteroatoms, e.g., B [16], N [17], P [18] and S [19]. These heteroatoms can modulate the electronic structure and surface properties of carbon-based materials. Among them, the nitrogen atoms are close to carbon atoms in the periodic table, making an ideal candidate for substituted in carbon-based materials. The highly electronegative N atoms as donors of electrons can donate electrons to alter the π-conjugated system and electron distribution of carbon materials, destroy their electrical neutrality, resulting in the improvement of electronic conductivity and material stability, optimization of surface wettability, promotion of charge transfer, increase in electrical capacity, and ultimately enhancing energy density [20]. According to their bonding configuration, it is well known that the introduction of nitrogen atoms is classified as pyridinic N, pyrrolic N, graphitic N, and N oxides [21], improving the properties of these materials or imparting new functionalities for applications such as CO_2_ adsorption and supercapacitors.

Currently, the introduction of nitrogen atoms in the porous carbon skeleton is crucially important for obtaining porous N-doped carbon spheres. According to the strategy for nitrogen atoms, two main methods have been employed for the preparation of N-doped porous carbon spheres, the first of which relies on the post-treatment of carbons with ammonia, urea and acetonitrile at high temperatures [22] and exhibits the drawbacks of high processing costs, low N contents and equipment corrosion. The second type corresponds to in situ doping using different N sources, including biomass, waste products, commercially available monomers (melamine, pyrrole, acetonitrile and ionic liquids) [23] and polymers (polypyrrole, phenolic resol and polydopamine) [24,25,26,27]. Techniques of this type rely on various design strategies, including templating, chemical vapour deposition, modified Stöber and hydrothermal methods and direct self-polymerisation. Compared to the post-treatment route, the in situ approach has been commonly used for the synthesis of nitrogen atom porous carbon spheres due to more straightforward, its simpler process, higher nitrogen content, and greater stability of the corresponding products. Specially, the direct self-polymerization assembly method has recently attracted much researcher interest in virtue of its advantages for the fabrication of colloidal porous carbon spheres. Liu et al. [28] reported a solvent-mediated polymerization-induced self-assembly pathway to obtain a polyphenol precursor, which successfully prepared hierarchically structured meso-macroporous N-doped carbon spheres by the pyrolysis process. Zhao et al. [29] synthesized monodisperse ultrahigh nitrogen-content mesoporous carbon nanospheres through an aqueous emulsion polymerization self-assembly approach, using melamine-formaldehyde resin oligomers as precursors. Meanwhile, nitrogen-doped carbon nanospheres with uniform sizes have been synthesized using phenolic resin oligomers as precursors and EDTA as stabilizing agents by Jacob Jeskey and co-workers [30]. Polydopamine can also be used as a source of N, e.g., Tang et al. fabricated mesoporous carbon nanospheres possessing a high nitrogen content using a soft-templating method by blending a diblock copolymer (PEO-b-PS) with polydopamine, followed by carbonization at 800 °C [31]. According to the above studies, it is found that the key points in the synthesis of porous N-doped carbon spheres through the direct self-polymerization assembly method mainly rely on organic–organic self-assembly between polymer monomers and block copolymers or organic agents, and manipulated interfacial interactions. Despite the progress in the synthesis of porous carbon spheres with diverse pore structures and morphologies, the simultaneous control of product morphology and hierarchical pore architecture is challenging. Therefore, the manipulation of interfacial interactions during the assembly process is essential for precisely controlling the morphology and pore structure of carbon spheres. To the best of our knowledge, few reports have described the controlled synthesis of hierarchical porous N-doped carbon spheres using mixed block copolymers as templates, and the synthesis of such spheres with well-integrated microporous/mesoporous architectures remains challenging.

Herein, we present the synthesis of N-doped hierarchically porous structured carbon spheres with a tunable configuration via the direct self-polymerization assembly method. The key point in this strategy is to build a micelle system by the combination of block copolymers as templates, 1,3,5-trimethylbenzene (TMB) and dopamine in the water/ethanol mixture. Furthermore, the assembly behaviour of the micelle system was controlled by the varying the hydrophobic/hydrophilic molar ratio of the block copolymer. Specifically, by adjusting hydrophobic/hydrophilic ratios of the block copolymer template from F127 (PEO_106_PPO_70_PEO_106_) to the composite template of F127/P123 and P123 (PEO_20_-PPO_70_-PEO_20_) [PEO: poly(ethylene oxide), PPO: poly(propylene oxide)], the micelle structure and interfacial interactions as well as the pore architectures and morphologies of the resultant carbon spheres underwent substantial transformations. Finally, the resultant nitrogen-doped carbon spheres presented diverse morphologies, including large hollow spherical shape, small hollow spherical shape, monodispersed regular spherical shape, and aggregated spherical shape. The resultant carbon spheres featured a hierarchically micro–mesoporous structure with micropores (~0.2/1.5 nm) and mesopores (∼2/10 nm), along with a high surface area (∼212/436 m^2^/g and N content (>3.0 wt%), showing the excellent CO_2_ adsorption capability and performance as supercapacitor electrode materials.

## 2. Results and Discussion

### 2.1. Structure and Morphology

#### 2.1.1. X-Ray Diffraction (XRD) Analysis

**Figure 1:**
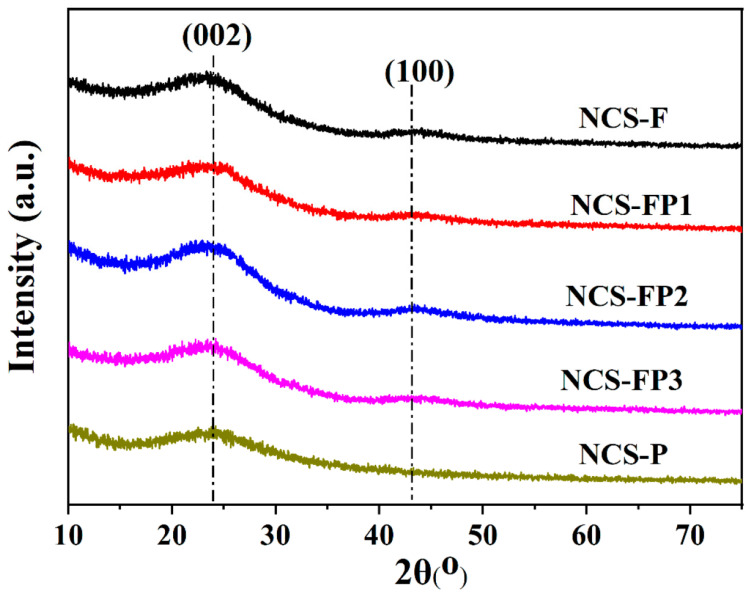
XRD patterns of NCS-F, NCS-FP1, NCS-FP2, NCS-FP3 and NCS-P.

Figure 1 shows the XRD patterns of samples for NCS-F, NCS-FP1, NCS-FP2, NCS-FP3 and NCS-P. For all samples, the two obvious diffraction peaks near 23.4° and 44.0° were observed, corresponding to the (002) and (001) lattice planes, respectively [32]. The results indicated the existence of a graphite phase for the N-doped mesoporous carbon spheres, which was calcinated at a higher temperature of 800 °C. According to Bragg’s law, the averaged d_(002)_ was calculated to be 3.79 Å, exceeding that of the natural graphite (3.35 Å), and thus a certain amorphousness. Furthermore, the nitrogen doping in the carbon material can cause an increase in interlayer distance of the carbon, which confirms the presence of structural nitrogen [33].

#### 2.1.2. Morphology and Surface Elemental Composition Analyses

**Figure 2:**
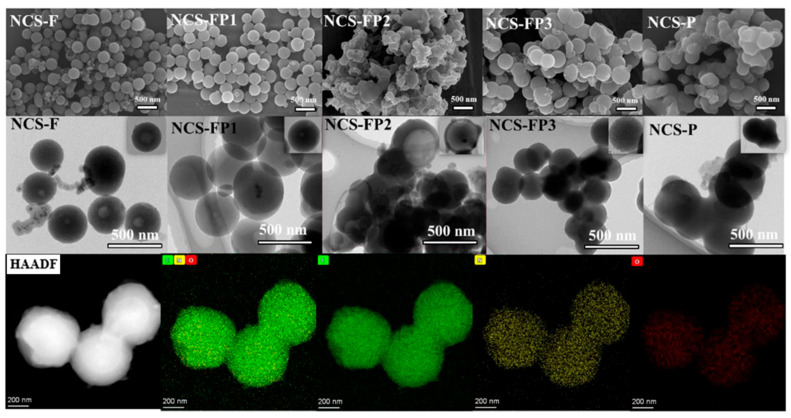
SEM and TEM images of NCS-F, NCS-FP1, NCS-FP2, NCS-FP3 and NCS-P and corresponding NCS-FP3 scanning TEM (C, N, O) energy-dispersive X-ray element mapping.

The microstructures of samples were characterized by SEM and TEM. As shown in Figure 2, the N-doped carbon spheres of NCS-F synthesised using F127 as a soft template contained monodispersed particles with an average particle size of ~700 nm and partially exhibited hollow morphologies. The magnified TEM image of NCS-F (Figure 2) confirms the monodisperse hollow structure of these particles. Meanwhile, to investigate the effect of template on the morphology of carbon spheres, NCS-FP1, NCS-FP2 and NCS-FP3 were synthesized using a mixture template of F127 and P123 as a soft template and measured by SEM and TEM, as shown in Figure 2. With of the increase in the hydrophobic/hydrophilic molar ratio of the block copolymer P123 template, the morphology of carbon spheres underwent significant alterations. For the NCS-FP1sample, the carbon spheres remained the smooth and monodispersed, while the hollow structure at the center of carbon spheres became smaller, as shown in Figure 2. In the case of the NCS-FP2 sample, the aggregated carbon spheres were generated with the larger cavity structure. Compared with NCS-FP2, NCS-FP3 prepared at an F127:P123 molar ratio of 1:3 exhibited a more uniform structure without cavities (Figure 2). Moreover, the N-doped carbon spheres for NCS-P, synthesized with P123 as a template, exhibited aggregated spherical particles with an average size of ~500 nm and no hollow structures. The internal structural diversity of the carbon spheres is further detailed in the insets of Figure 2. These results demonstrated that the variation hydrophobic/hydrophilic molar ratio of the block copolymer templates could be used to adjust the micellar morphology and thus control product microstructure. According to Zhao [34], as the volume of hydrophobic [poly (propylene oxide)] and hydrophilic [poly (ethylene oxide)] increased, the micellar structure gradually evolved from spherical to lamellar, enabling the possibility to continuously tune pore structures.

In this system, F127, P123, DA and TMB assembled to form stable spherical structures with precursor molar ratio-dependent interfacial curvatures. The micelle structure is related to the surfactant packing parameter *P*, which, in turn, is positively correlated with the hydrophobic (PPO)/hydrophilic (PEO) volume ratio. Higher *p* values cause a micelle structure change from spherical to other shapes and may even prevent micelle formation. Based on the abovementioned results, when the hydrophobic (PPO)/hydrophilic (PEO) volume ratio was low (0.45 for F127), the highly hydrophilic PEO chains formed a stacked state in the water–alcohol system and effectively self-assembled with dopamine and TMB to form a single-cavity structure. When the above volume ratio increased (*n*_F127_/*n*_P123_ = 3:1, 0.48), F127 still dominated and formed an effective self-assembly structure. The micelle hydrophobicity increased, and the interfacial curvature minimally decreased, which lowered the abundance of the hollow structures. When the above volume ratio reached 0.79 (*n*_F127_/*n*_P123_ = 1:1), the two surfactants did not form a uniformly ordered micelle structure, which resulted in the formation of more aggregated particles. Finally, when the volume ratio exceeded 1.0 (1.15 for *n*_F127_/*n*_P123_= 1:3 and 4.8 for P123), corresponding to a high hydrophobicity, *P* increased, the micelle structure changed, and the interfacial curvature decreased. Under these conditions, an effective self-assembly structure formed, resulting in spherical particles without cavities.

NCS-FP3 exhibited uniform C, N and O distributions (Figure 2).

#### 2.1.3. Surface Area and Pore Structure Analyses

**Figure 3:**
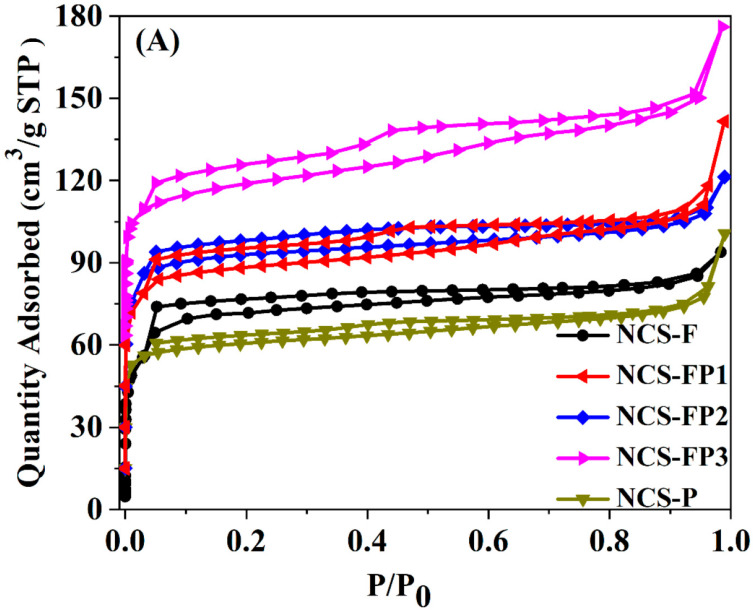

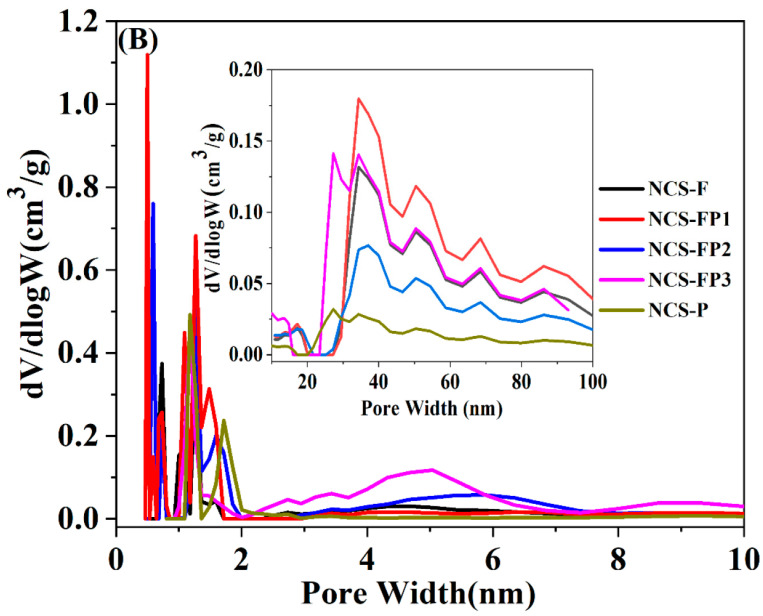
N_2_ sorption isotherms (**A**) of NCS-F, NCS-FP1, NCS-FP2, NCS-FP3 and NCS-P and the corresponding pore size distribution curves (**B**).

Figure 3A shows the N_2_ adsorption–desorption isotherms, with the corresponding textural parameters summarized in Table 1. As shown in Figure 3A, all sorption isotherms had Type Ι and Type IV features, indicating the presence of both micropores and mesopores [35]. Specifically, for all samples, the nitrogen adsorption capacity rapidly increased at the low relative pressure (*p*/*p*_0_ < 0.01), indicating the existence of microporous structure. Meanwhile, the capillary condensation of liquid nitrogen in mesopores in the relative pressure range of 0.03–0.99 was observed. The slight increase in nitrogen adsorption capacity at high relative pressures (*p*/*p*_0_ > 0.95) suggested the formation of macropores. In addition, the pore-size distribution for all samples was obtained using the density functional theory (DFT) method, as shown in Figure 3B. The peaks of pore size diameter for all samples were mainly in the ranges of 0.5–1.0, 1.0–1.3, 1.3–2.0, 2.0–7.0 and 20–100 nm, further verifying the existence of hierarchical porous structures (micropore, mesopore and macropore). The hierarchical porous structure favoured for material transformation and working as adsorbent and electrode material.

As shown in Table 1, the specific surface areas of NCS-F, NCS-FP1, NCS-FP2, NCS-FP3, and NCS-P were calculated to be 238, 353, 369, 463 and 224 m^2^/g, respectively, and total pore volumes of 0.16, 0.22, 0.19, 0.27 and 0.11 cm^3^/g, respectively. The relative micropore content exceeded 50% in all cases, indicating the dominance of microporous structures. The surface area with the increasing content of P123 in the composite template (see data for NCS-FP1, NCS-FP2 and NCS-FP3) was attributed to the interaction between the two surfactants as structure-directing agents during the self-assembly process involving PDA and TMB. At the microscopic level, molecules are likely to interweave and entangle with each other, which increases the degree of disorder and favours the formation of additional pore structures and, hence, high specific surface areas. This hypothesis was validated using small-angle XRD measurements (Figure S1). The patterns of NCS-F and NCS-P featured broad peaks at 2*θ* = 0.8–1.5°, implying the existence of an orderly porous structure. This was not the case for NCS-FP1, NCS-FP2 and NCS-FP3, which indicated that the F127–P123 composite templates could have affected the ordered arrangement of the micelles and were more likely to afford a more open pore structure leading to a higher surface area.

These findings demonstrate that the hydrophobic/hydrophilic molar ratio of the molecular chains of block copolymer surfactants plays a critical role in determining the morphology and structure of the end product, in line with the results of the related SEM and TEM analyses.

#### 2.1.4. Fourier-Transform Infrared (FTIR) Analysis

**Figure 4:**
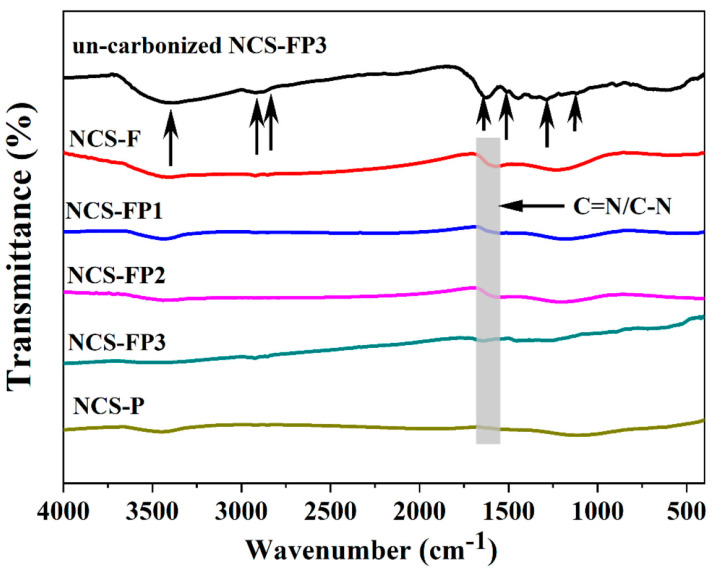
FT-IR spectra of NCS-F, NCS-FP1, NCS-FP2, NCS-FP3, NCS-P and un-carbonized NCS-FP3 (The arrows represent the peak positions corresponding to the functional groups.)

The FTIR absorption spectra of carbonised samples and noncarbonised PDA in NCS-FP3 are shown in Figure 4. The spectrum of noncarbonised PDA in NCS-FP3 exhibited a band at 3396 cm^−1^, which was assigned to -OH and -NH_2_ groups. The bands at 2932 and 2830 cm^−1^ were ascribed to the antisymmetric and symmetric stretching vibrations of the -CH_2_- groups of PDA, respectively [36]. The sharp peaks at 1612, 1496, 1288 and 1102 cm^−1^ were ascribed to C=O, -NH_2_, C-O and C-O-C moieties, respectively [37,38,39]. These results confirm the presence of nitrogenated functional groups. The catechol and amino groups of dopamine can be oxidised to quinone and secondary amine groups, respectively [24]. After carbonisation, the absorption peaks of the PDA-derived moieties substantially weakened, which indicated that PDA was successfully carbonised. The new absorption band at 1600 ± 20 cm^−1^ was assigned to C=N/C-N groups [37]. This result indicates that various N species were retained within the carbon matrix after high-temperature carbonisation, as supported by the X-ray photoelectron spectroscopy (XPS) data provided later in the text.

#### 2.1.5. Raman Analysis

**Figure 5:**
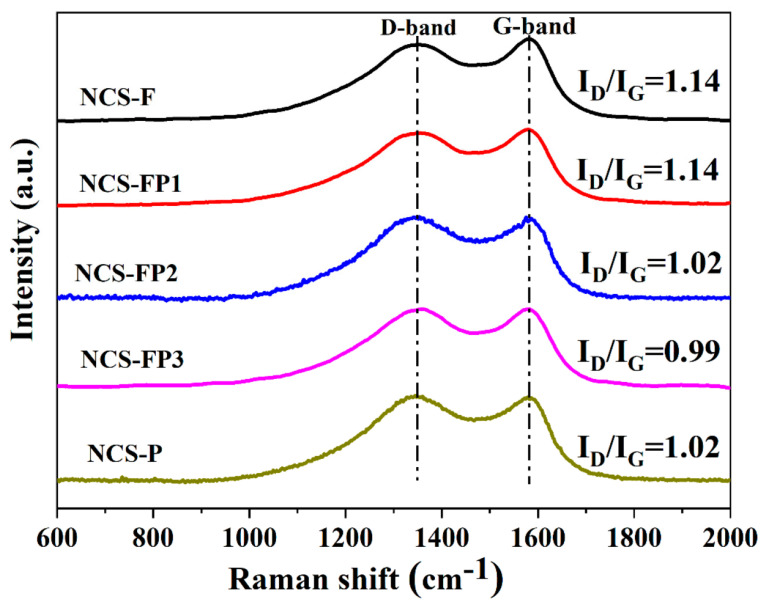
Raman spectra of NCS-F, NCS-FP1, NCS-FP2, NCS-FP3, and NCS-P.

The carbon structure of the samples was probed by Raman spectroscopy (Figure 5). The Raman spectra featured disordered carbon (D) and graphite (G) bands at 1348 and 1583 cm^−1^, respectively. The D band corresponded to the defects and disorder generated through N doping and the dangling bonds at the carbon edges produced during carbonisation. The G band corresponded to the in-plane vibration of sp^2^ hybridised carbons, suggesting the existence of a typical graphite structure. The D-to-G band intensity ratio (*I*_D_/*I*_G_) is negatively correlated with the degree of graphitisation and therefore often used to evaluate the same [40]. The *I*_D_/*I*_G_ ratios of NCS-F, NCS-FP1, NCS-FP2 and NCS-P (1.02–1.14) indicated the presence of defects and amorphous structure of the porous carbon spheres, in agreement with the XRD results. NCS-FP3 featured the lowest *I*_D_/*I*_G_ ratio (0.99) and, hence, the highest degree of graphitisation. The electrical conductivity of carbon materials can be improved through graphitisation.

#### 2.1.6. XPS Analysis

**Figure 6:**
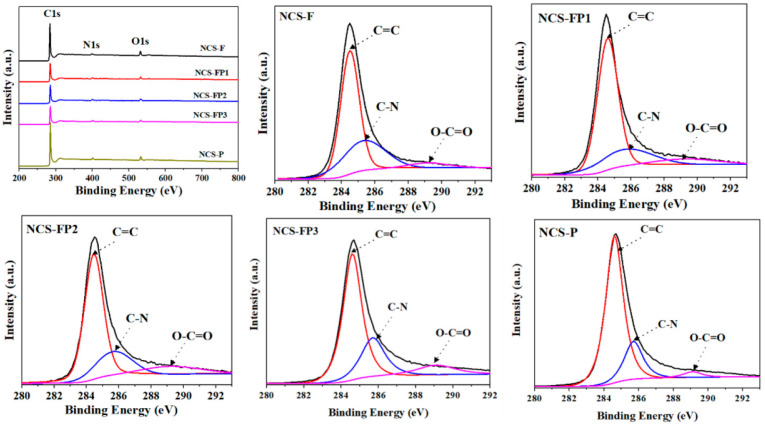

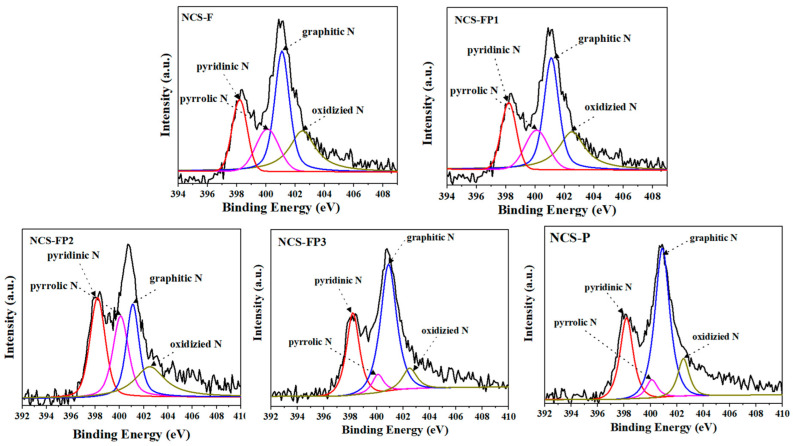
XPS survey spectra and high-resolution XPS spectra of C 1s and N 1s of NCS-F, NCS-FP1, NCS-FP2, NCS-FP3 and NCS-P (The different coloured curves represent the fitted peak curves).

The survey and high-resolution (C 1s and N 1s) X-ray photoelectron spectra of the samples are shown in Figure 6. C, N and O were detected in all samples, indicating the successful synthesis of N-doped carbon materials. The surface compositions determined by XPS are listed in Table 2. The C 1s spectra showed peaks at 284.6 (C=C), 285.7 (C=N) and 289.2 (O-C=O) eV. The N 1s spectra were deconvoluted into the peaks of pyridinic (398.2 eV), pyrrolic (400.1 eV), graphitic (401.1 eV) and oxidised (402.50 eV) N [29]. This result reveals that N in self-assembled polydopamine after high-temperature carbonisation was transformed into various nitrogenated species. According to previous studies, pyridinic and pyrrolic N can act as active sites and thus enhance electrochemical performance [41,42], mainly because the negatively charged lone electron pairs of pyridinic N can engage in reversible redox reactions with electrolyte ions in both acidic and alkaline media to generate pseudocapacitance. Additionally, graphitic and oxidised N can function as electron acceptors to improve the conductivity of carbon electrodes during charge–discharge and thereby reduce their ion transfer resistance [43]. Moreover, pyridinic N is Lewis basic and therefore favours CO_2_ adsorption [44]. After high-temperature carbonisation, the N content decreased from 9.15 wt% (PDA) to 3.88-4.85 wt% (hierarchical porous N-doped carbon spheres). This result indicates that polydopamine experienced a certain N loss under high-temperature conditions. The relative contents of the four types of N extracted from the deconvoluted N 1s spectra are listed in Table 2. NCS-FP2 and NCS-FP3 exhibited the highest N contents and therefore featured the highest CO_2_ capture and pseudocapacitance performances. The reference data from relevant studies used to analyse the FTIR and X-ray photoelectron spectra are listed in Tables S1 and S2, respectively.

### 2.2. CO_2_ Adsorption Performance

**Figure 7:**
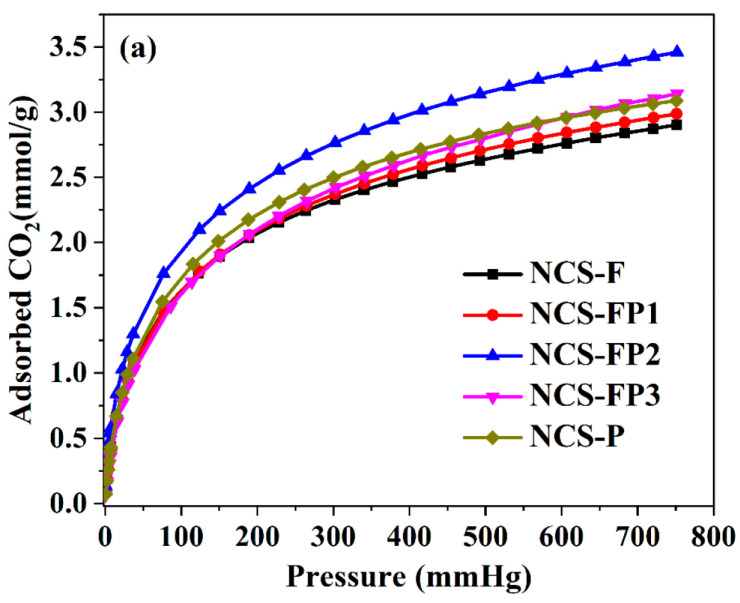

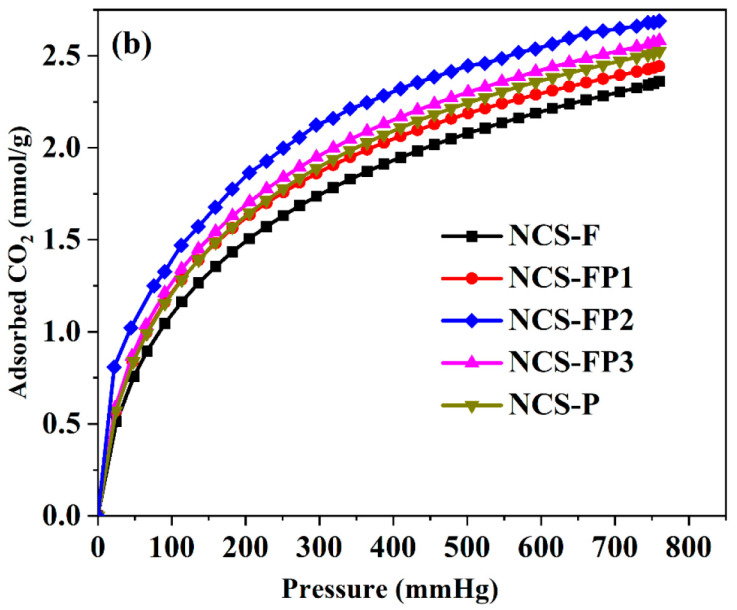
CO_2_ adsorption isotherms of NCS-F, NCS-FP1, NCS-FP2, NCS-FP3 and NCS-P (**a**) at 273 K and (**b**) at 298 K.

Figure 7 shows the CO_2_ adsorption isotherms of different samples, and Table 1 lists their CO_2_ adsorption capacities at 273 and 298 K. All samples exhibited high CO_2_ capture capacities, which were closely correlated with the surface area and pore size distribution. At 273 K and 760 mmHg, the CO_2_ capture capacities of NCS-F, NCS-FP1, NCS-FP2, NCS-FP3 and NCS-FP equalled 2.90, 2.98, 3.46, 3.15 and 3.08 mmol/g, respectively. The CO_2_ capture capacities of NCS-FP2, NCS-FP3 and NCS-P exceeded those of NCS-F and NCS-FP1. This finding was attributed to the well-developed microporous structures of NCS-FP2, NCS-FP3 and NCS-P, which featured relative micropore contents (volume-based) of 60.2%, 53.7% and 52.1%, respectively. Furthermore, although NCS-FP3 exhibited a more developed pore structure and higher surface area than NCS-FP2, the CO_2_ capture capacity of the former was lower because of its lower micropore content (53.7%). As reported previously [45], the microporous structure enhanced the CO_2_ capture capability, enabling strong bonding between the CO_2_ molecules and adsorbent in the micropores and promoting the packing of gas molecules [46].

NCS-FP2 exhibited the highest CO_2_ capture capacity, which was attributed to its highest N content (4.58 wt% based on XPS data). Nitrogenated groups can undergo acid–base reactions with CO_2_ molecules. In addition, hydrogen bonds can be formed between the carbon matrix surface and CO_2_ molecules [47]. The lower CO_2_ adsorption capacity of NCS-FP1 compared with those of the other samples may also be attributed to the lower N content of the former. Thus, high N contents favoured CO_2_ capture. As reported elsewhere (Table 3), non-N-doped carbon materials exhibit lower CO_2_ adsorption capacities than N-doped ones, which indicates that the introduction of N into the carbon matrix increases the CO_2_ adsorption capacity. However, as shown in Table 3, the CO_2_ adsorption capacity does not have a particular relation with the N content and specific surface area. Based on the above, we concluded that CO_2_ capture capability was due to the synergistic effects of the pore structure (pore volume and surface area) and surface chemistry features (nitrogenated functional groups). The enhanced micropore volume facilitates CO_2_ diffusion into the inner micropores, increasing the interaction energy with the adsorbent and improving gas molecule packing. Additionally, N doping and hydrogen bonding in the carbon matrix enhance basicity, thereby favouring the binding of the acidic CO_2_.

Upon a temperature increase to 298 K, the CO_2_ adsorption capacity decreased in all cases (Figure 7b and Table 1), equalling 2.36 mmol/g for NCS-F, 2.44 mmol/g for NCS-FP1, 2.69 mmol/g for NCS-FP2, 2.58 mmol/g for NCS-FP3 and 2.52 mmol/g for NCS-P. Thus, CO_2_ adsorption was concluded to be exothermic.

### 2.3. Electrochemical Performance

**Figure 8:**
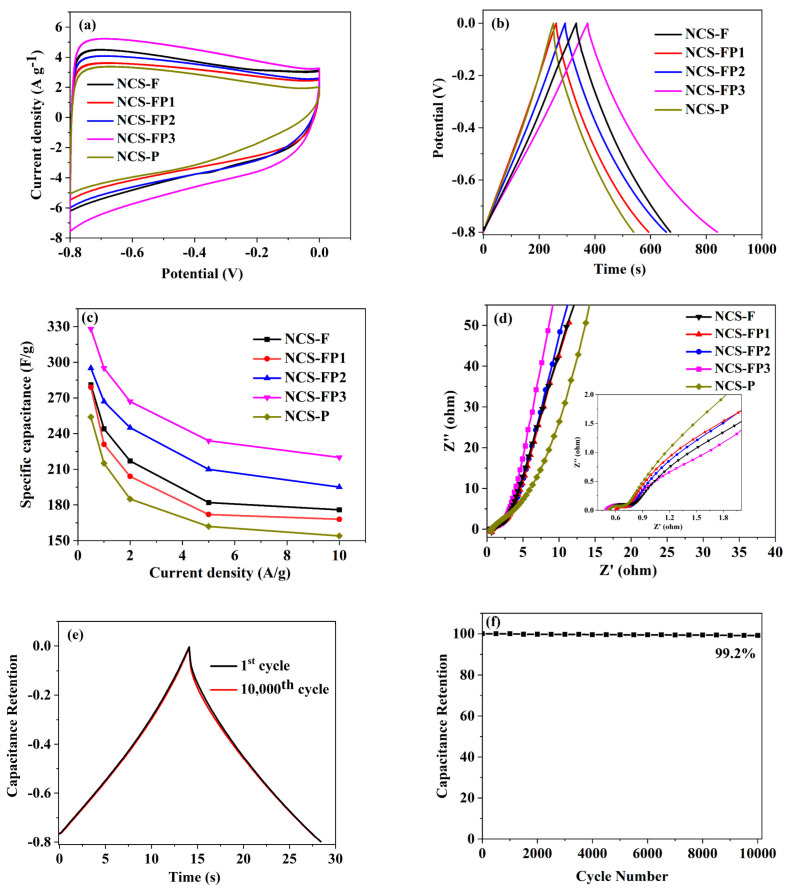
Electrochemical evaluation of the N-doped porous carbon sphere: (**a**) CV curves of different samples at 10 mV/s; (**b**) GCD curves of different samples at 0.5 A/g; (**c**) specific capacitances at different GCD current densities; (**d**) Nyquist plots with fitting curves and their corresponding high frequency ranges (inset); (**e**,**f**) cycling stability of NCS-FP3.

Figure 8a shows the cyclic voltammetry (CV) curves for these sample electrodes within a potential window of −0.8 to 0 V at a scan rate of 10 mV/s. As can be seen from the figure, the CV curves for all samples displayed a transformative rectangular-like shape, due to the coexistence of the double-layer capacitor and pseudo capacitance. This behaviour was attributed to the oxidation–reduction reactions arising from the N, O co-doping. Moreover, the NCS-FP3 electrode demonstrated a significantly larger integrated area under its CV curve than the NCS-F, NCS FP1, NCS-FP2 and NCS-P electrodes, primarily because of its higher surface area and hierarchical porous structure. According to previously reported studies, mesopores can supply the channels of ion transfer and favour the storage of high energy. In order to verify the results obtained by the CV test, the corresponding galvanostatic charge–discharge (GCD) measurement was also carried out for these N-doped porous carbon spheres. Figure 8b shows the GCD curves of all samples at a low current density of 0.5 A/g. The GCD curves present highly symmetrical and isosceles triangular shapes, implying excellent electrochemical reversibility of the electrodes. NCS-FP3 exhibited the longest charging time. Furthermore, based on the obtained GCD profiles, the variation trend of specific capacitance as a function of current density (ranging from 0.5 to 10 A/g) was observed, as shown in Figure 8c. The specific capacitances of NCS-F, NCS-FP1, NCS-FP2, NCS-FP3 and NCS-P at 0.5 A/g equalled 282.6, 278.6, 294.2, 328.3 and 254.2 F/g, respectively, decreasing to 175.3, 167.4, 194.8, 219.5 and 153.6 F/g, respectively, at 10 A/g, which corresponded to retention rates of 62.0%, 60.0%, 66.2%, 66.9% and 60.4%, respectively, at a current density range of 0.5–10 A/g. NCS-FP3 showed the highest specific capacitance and excellent capacitance retentions, because of its large surface area, N doping, and hierarchical porous structure with micropores and mesopores. The mesoporous structure facilitates the rapid electrolyte transport towards the micropores, thereby enhancing the charge storage capacity. These results are consistent with those of CV tests.

To investigate the conductivity and diffusion behaviour of these N-doped carbon spheres, electrochemical impedance tests (EIS) were performed, and the relevant Nyquist plots are shown in Figure 8d. The Nyquist plot for all samples displayed an approximately vertical line in the low frequence region, implying an excellent capacitance behaviour. The equivalent series resistance of these N-doping carbon spheres can be evaluated from the *x*-intercepts of the corresponding Nyquist plots as 0.53, 0.61, 0.59, 0.49 and 0.51 Ω, respectively. For NCS-FP3, the Nyquist plot was the closest to the imaginary axis (−Z″), indicating a faster ion and electron transfer during the capacitance response compared to the other samples. This enhanced performance was attributed to its higher surface area, N doping, and hierarchical porous structure of NCS-FP3. The high surface area can improve the contact area between the electrode and electrolyte interface, facilitating better charge storage and transport. In the meantime, it can be clearly seen in Figure 8d(inset) that the Nyquist plots for all samples presented a small semicircle in the high frequency region, implying a low charge-transfer resistance (R_ct_). To evaluate the cycling stability, the NCF-FP3 sample was measured for 10,000 cycles at a current density of 10 A/g, as shown in Figure 8e. The capacitance retention remained 99.2% of its original capacitance after 10,000 cycles. Nyquist plots before and after 10,000 cycles (Figure 8f) revealed that the series resistance (Rs) of the NCF-FP3 electrode remains essentially unchanged after cycling, indicating its good stability. Table 4 compares the supercapacitive performance of N-doped porous carbon spheres with those of recently reported materials. As shown in Table 4, despite variations in the preparation methods, structure, and surface specific area of N-doping carbon spheres reported in related literature, NCS-FP3 presented a notably higher specific capacitance and GCD cycling stability compared to most N-doping carbon spheres reported, demonstrating its potential as an electrode material in supercapacitors.

## 3. Experimental Section

### 3.1. Materials

DA Dopamine hydrochloride (C_8_H_11_NO_2_·HCl 98 wt%), F127 (PEO_106_PPO_70_PEO_106_ *M*_W_ = 12,600), and Pluronic P123 (PEO_20_-PPO_70_-PEO_20_ *M*_W_ = 5800) were supplied by Aladdin Co. Ltd. (Shanghai, China). TMB 1,3,5-trimethylbenzene (C_9_H_12_) was provided by Shanghai Maclin Biochemical Technology Co., Ltd. (Shanghai, China). Aqueous ammonia (NH_3_·H_2_O, 28–30 wt%) was purchased from Tianjin Da Yong Chemical Reagent Co., Ltd. (Tianjin, China), and ethanol (C_2_H_6_O) was obtained from Sinopharm Chemical Reagent Co. Ltd. (Shanghai, China). 

### 3.2. Preparation of Hierarchical Porous N-Doped Carbon Spheres

The typical synthesis procedure of N-doped hierarchically porous spheres is as follows: 0.6 g F127 as a template was dissolved in a mixture of 18 mL ethanol and 18 mL distilled water upon stirring at 400 rpm at room temperature. The mixture was supplemented with TMB (1.0 mL), stirred for 1.5 h at room temperature, supplemented with dopamine hydrochloride (0.6 g) upon continuous stirring and then dropwise supplemented with aqueous ammonia (1.0 mL, basic catalyst) to promote DA polymerisation. The mixture was stirred for 24 h at room temperature. The obtained black precipitation, which served as an intermediate product of N-doped hierarchical porous carbon sphere, was collected by centrifugation after 10 min at 8000 rpm and subsequently washed several times with ethanol and water. The resulting black solid was dried for 12 h at 100 °C. The final products were heated at 350 °C for 3 h and subsequently heated at 800 °C for 2 h under a nitrogen atmosphere at a heating rate of 2.0 °C/min. To tailor the morphology and porous structure of the N-doped hierarchical pore carbon spheres, the molar ratio of F127 to P123 templates was altered as follows: 1:0, 3:1, 1:1, 1:3, and 0:1. The resulting samples were denoted as NCS-F, NCS-FP1, NCS-FP2, NCS-FP3 and NCS-P, respectively, and collectively referred to as NCS-*x*.

### 3.3. Characterisation

The scanning electron microscopy (SEM) images were obtained using a Zeiss Sigma 300 electron microscope at an acceleration voltage of 15 kV (Zeiss, Jena, Germany). The TEM micrograph was produced by FEI Talos 200S at 200 kV, which is equipped with transmission electron microscopy (TEM) and energy dispersive spectroscopy (EDS) detectors for element mapping analysis. The powder X-ray diffraction (XRD) measurements were recorded on a Rigaku SmartLab SE diffractometer (Rigaku, Tokyo, Japan) using Cu *Kα* radiation (*λ*= 0.154056 nm) source in 2θ ranges from 10.0° to 80.0° with a scanning speed of 5.0 °/min and operating at 35 KV and 20 mA. Fourier-transform infrared (FT-IR) spectra were measured on a Nicolet iS10 analyzer with a scanned range of 4000–400 cm^−1^ and a resolution of 4 cm^−1^. Surface area and porosity were determined from the nitrogen adsorption–desorption isotherm obtained (at 77 K) using a micromeritics ASAP 3020 sorptionmeter (Micromeritics, Norcross, GA, USA). The N-doped hierarchical porous carbon spheres were pre-treated at 150 °C for 8 h under a nitrogen atmosphere. The isotherm data were analysed by the BET (Brunauer–Emment–Teller) method, and the pore size distributions were calculated using density functional theory (DFT). X-ray photoelectron spectroscopy (XPS) was recorded on a Thermo Scientific K-Alpha spectrometer with Al anode *Kα* radiation (*hv* = 1486.6 eV). Carbonaceous C 1s line (284.8 eV) was used as a reference to calibrate the binding energies. The Raman spectra were collected on Horiba Scientific LabRAM HR Evolution (Horiba, Kyoto, Japan) with 532.05 nm incident radiation.

### 3.4. CO_2_ Adsorption Performance Evaluation

CO_2_ (99.99%) adsorption isotherms (Micromeritics TriStar II 3fle, Micromeritics, Norcross, GA, USA) were recorded at 273 and 298 K in a pressure range of 0–760 mmHg. The temperatures of 273 K were maintained using an ice–water bath and a water recirculator. Prior to each adsorption experiment, the samples (~100 mg) were degassed for 8 h at 180 °C in a flow of N_2_ (99.999%, 100 mL/min) to remove guest molecules from the pores. Subsequently, the samples were cooled to the desired temperature, and pure CO_2_ was introduced into the system. CO_2_ adsorption was performed in situ using a working station.

### 3.5. Electrochemical Testing

Electrochemical measurements were performed using a three-electrode system in 1.0 M KOH electrolytes. The electrode system contained Hg/HgO as a reference electrode, Pt sheet as the counter electrode, and NCS-*x* as the working electrode. To prepare the working electrode, mesoporous carbon, commercial acetylene black and polytetrafluoroethylene were mixed in a mass ratio of 8:1:1 and ground for 3 h with ethanol as a wetting agent in an agate mortar. The powder block was then pressed on Ni foam with approximate dimensions of 1 cm × 1 cm and dried at 100 °C in 12 h in a vacuum oven. The carbon loading of the electrode was 3–4 mg/cm^2^. Electrochemical tests (CV, GCD, EIS and cycling stability measurements) were conducted using a CHI 760E electrochemical workstation (CH Instruments, Bee Cave, TX, USA). Specific capacitance (*C*_g_, F/g) was calculated as follows:

(1) $$Cg\;=\frac{I\Delta t}{m\Delta V}$$

where *I*, Δ*t*, Δ*V*, and *m* represent for the discharge current (A), the discharge time (s), the working voltage window and the mass of actual active materials in the work electrode, respectively.

## 4. Conclusions

In summary, the direct self-polymerization assembly method has been developed for the synthesis of N-doped hierarchical porous carbon spheres by using F127, F127/P123 and P123 as templates and dopamine as the precursor. A stable micellar system was formed through the combination of block copolymers, TMB, and dopamine in a water/ethanol mixture. The micellar structure in this synthesis was tuned by the regulating hydrophobic/hydrophilic ratio of the surfactants, which resulted in the controllable porous structures and morphology including single hollow spheres, monodispersed regular spheres, and aggregated spheres. The resulting porous N-doped carbon spheres exhibited a hierarchical porous structure with micropores (~0.2/1.5 nm) and mesopores (~2/10 nm), a high surface area (~224/436 m^2^/g) and N content (~3.77/4.58 wt%) and outstanding CO_2_ adsorption capacities (2.90–3.46 mmol/g) at 273 K/760 mmHg, and (2.36–2.69 mmol/g) at 298 K/760 mmHg. Notably, the resultant NCS-FP3 showed the best electrochemical performance as a supercapacitor electrode material, which performed high specific capacitance (328.3 F/g) at 0.5 A/g and excellent electrochemical cycling stability (99.2% retention after cycling 10,000 times at 10 A/g) and outperformed most previously reported porous carbon spheres owing to its high surface area, N doping and hierarchical porous structure featuring both micropores and mesopores. The mesoporous structure facilitated rapid electrolyte transport towards micropores, thereby enhancing charge storage capacity. These results indicated that the obtained N-doped hierarchical porous carbon spheres by the direct self-polymerization assembly method hold significant potential for application as CO_2_ capture and supercapacitor electrodes.

## Figures and Tables

**Table 1:** The structure parameters of all related samples.

Samples	BET Surface Area ^a^ (m^2^/g)	V_micro_^b^ (cm^3^/g)	V_total_^c^ (cm^3^/g)	V_micro_/V_total_ (%)	Average Pore Diameter ^d^ (nm)	CO_2_ Uptake ^e^ (mmol/g)
NCS-F	238	0.08	0.16	50.2	11.1	2.90/2.36
NCS-FP1	353	0.11	0.22	51.6	9.2	2.98/2.44
NCS-FP2	369	0.12	0.19	60.2	10.0	3.46/2.69
NCS-FP3	463	0.15	0.27	53.7	7.05	3.15/2.58
NCS-P	224	0.06	0.11	52.1	6.05	3.08/2.52

^a^ Calculated by the BrunauerEmmett-Teller (BET) surface area. ^b^ Calculated by T-plot model. ^c^ Estimated from the adsorbed amount of nitrogen at *p*/*p*_0_ > 0.995. ^d^ Calculated by the BJH model from the adsorption branches of the isotherms. ^e^ CO_2_ uptake at 273 K/298 K and 760 mmHg.

**Table 2:** Surface contents of C, N, O elements and concentrations ratios of the nitrogen species (wt%) obtained from XPS spectra.

Samples	C	N	O	Pyridinic-N	Pyrrolic-N	Graphitic-N	Oxidized-N
NCS-F	88.73	3.88	7.39	0.85	0.75	1.38	0.85
NCS-FP1	88.47	3.77	7.76	0.78	0.72	1.39	0.85
NCS-FP2	88.49	4.58	7.01	1.37	1.16	1.15	0.89
NCS-FP3	89.31	4.39	6.30	1.17	0.21	2.25	0.51
NCS-P	89.95	3.85	6.20	1.00	0.19	2.18	0.44

**Table 3:** CO_2_ adsorption performance of porous carbon spheres.

**Sample**	**S_BET_ (m^2^/g)**	**Situation of N-Doped (XPS)**	**Test Condition**	**CO_2_ Uptake (mmol/g)**	**Ref.**
Activated Carbon	671	N-undoped	25 °C, 100% CO_2_, 1 bar	2.13	[48]
Derived carbon	717	N-undoped	0 °C, 100% CO_2_, 1 bar	1.62	[49]
Hollow Carbon sphere	1369	N-doped (1.53%)	0 °C, 100% CO_2_, 1 bar	2.63	[50]
Hollow carbon nanoparticles	1716	N-doped (3.82%)	0 °C, 100% CO_2_, 1 bar	5.11	[51]
Carbon microspheres	537.4	N-doped (5.34%)	0 °C, 100% CO_2_, 1 bar	3.46	[52]
Carbon Spheres/NCS-FP3	369	N-doped (4.03%)	0 °C, 100% CO_2_, 1 bar	3.46	This work

**Table 4:** Electrochemical performance of N-doping porous carbon sphere electrodes for supercapacitor applications.

Samples	S_BET(m_^2^_/g)_	Electrolyte	C_sp_ (F/g)	Stability/Cycles	Ref.
NCS_NH3_-950	1049	1 M H_2_SO_4_	244 (0.2 A/g)	87.6% (10,000)	[53]
N-MHCNs-2	430	6 M KOH	241 (0.5 A/g)	90.4% (3000)	[54]
N-MCS	717	6 M KOH	273 (0.5 A/g)	93.0% (10,000)	[55]
NHCSs	2044	EMIBF_4_	234 (0.5 A/g)	91.0% (20,000)	[56]
N-YSCS	1263	6 M KOH	242 (1.0 A/g)	97.3% (5000)	[57]
NIHCSs-2	702	6 M KOH	259 (1.0 A/g)	70.3% (10,000)	[58]
NYCs-800	780	6 M KOH	301 (1.0 A/g)	91.3% (10,000)	[59]
NPCSs-P1.5-85-4	1040	6 M KOH	181 (1.0 A/g)	96.0% (5000)	[60]
NCS-FP3	463	1 M KOH	328 (0.5 A/g)	99.2% (10,000)	This work

## Data Availability

All data included in this study are available upon request by contacting the corresponding author.

## Supplementary Materials

The following supporting information can be downloaded at https://www.mdpi.com/article/10.3390/molecules30132747/s1: Supplementary data: Figure S1. XRD patterns of samples; Table S1. The comparative tables of reference data of FT-IR; Table S2. The comparative tables of reference data of XPS.

## Abbreviations

The following abbreviation are used in this manuscript:

XRD	X-ray diffraction
SEM	Scanning electron microscopy
TEM	Transmission electron microscopy
EDS	Energy dispersive spectroscopy
FT-IR	Fourier-transform infrared
XPS	X-ray photoelectron spectroscopy
DFT	Density functional theory
CV	Cyclic voltammetry
GCD	Galvanostatic charge/discharge
EIS	Electrochemical impedance spectroscopy
R_ct_	Charge-transfer resistance
F127	Polyethylene oxide_(106)_-block-polypropylene oxide_(70)_-block-polyethylene oxide_(106)_
P123	Polyethylene oxide_(20)_-block-polypropylene oxide_(70)_-block-polyethylene oxide_(20)_
TMB	1,3,5-trimethylbenzene
Po	Polyoxyethylene
DA	Dopamine
PDA	Polydopamine
PTFE	Polytetrafluoroethylene
