# Supplementary material for: Controlled Synthesis of N-Doped Hierarchical Porous Carbon Spheres Through Polydopamine for CO2 Adsorption and High-Performance Supercapacitors
Source: Molecules. 2025 Jun 26;30(13):2747. doi: 10.3390/molecules30132747 (PMC12250799; doi:10.3390/molecules30132747)
Supplement: Supplementary file 1 [file molecules-30-02747-s001.zip › molecules-3646882-supplementary.pdf]

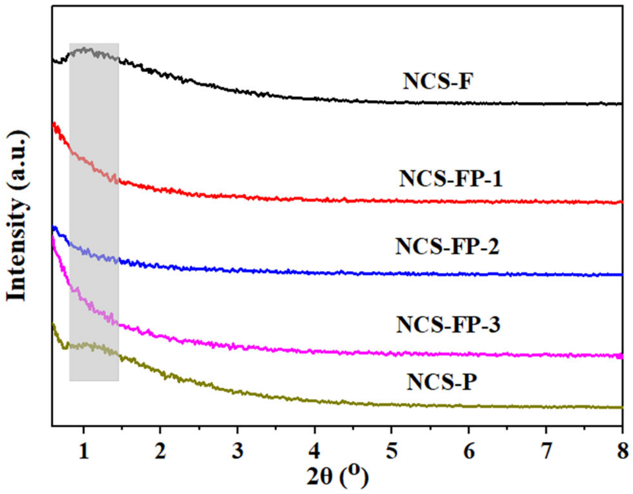

Fig. S1 XRD patterns of samples

Table S1. The comparative tables of reference data of FT-IR

Groups	$\nu_{\text{-OH/NH2}}$	$\nu_{\text{-CH2}}$	$\nu_{\text{C=O}}$	$\delta_{\text{-NH2}}$	$\nu_{\text{C-O}}$	$\nu_{\text{C-O-C}}$	$\nu_{\text{C=N/C-N}}$
Bands/ cm^{-1} This work	3396	2932 2830	1612	1496	1288	1102	1600±20
[36]	3395	2930/ 2835	—	—	—	—	—
[37]	—	—	—	—	—	1105	1600±20
[38]	3451	2928 2853	—	—	1260	1100	—
[39]	3108-3345	—	—	—	1285	1103	1608

Table S2. The comparative tables of reference data of XPS

Groups	C=C	C=N	O-C=O	Pyridinic-N	Pyrrolic-N	Graphitic-N	Oxidized-N
Peaks/ eV This work	284.6	285.7	289.2	398.2	400.1	401.1	402.50
[29]	284.6	285.7	289.1	398.4	400.1	401.1	402.5
